# Improving CRISPR–Cas9 directed faithful transgene integration outcomes by reducing unwanted random DNA integration

**DOI:** 10.1186/s12929-024-01020-x

**Published:** 2024-03-26

**Authors:** Rio Hermantara, Laura Richmond, Aqeel Faisal Taqi, Sabari Chilaka, Valentine Jeantet, Ileana Guerrini, Katherine West, Adam West

**Affiliations:** 1 School of Cancer Sciences, College of Medical, Veterinary and Life Sciences, University of Glasgow, Glasgow, UK; 2 Department of Biomedicine, School of Life Sciences, Indonesia International Institute for Life Sciences, Jakarta, Indonesia; 3 School of Molecular Biosciences, College of Medical, Veterinary and Life Sciences, University of Glasgow, Glasgow, UK

**Keywords:** CRISPR–Cas9, Knock-in, Off-target, Faithful genome editing, Self-cleaving, Homology arms

## Abstract

**Background:**

The field of genome editing has been revolutionized by the development of an easily programmable editing tool, the CRISPR–Cas9. Despite its promise, off-target activity of Cas9 posed a great disadvantage for genome editing purposes by causing DNA double strand breaks at off-target locations and causing unwanted editing outcomes. Furthermore, for gene integration applications, which introduce transgene sequences, integration of transgenes to off-target sites could be harmful, hard to detect, and reduce faithful genome editing efficiency.

**Method:**

Here we report the development of a multicolour fluorescence assay for studying CRISPR–Cas9-directed gene integration at an endogenous locus in human cell lines. We examine genetic integration of reporter genes in transiently transfected cells as well as puromycin-selected stable cell lines to determine the fidelity of multiple CRISPR–Cas9 strategies.

**Result:**

We found that there is a high occurrence of unwanted DNA integration which tarnished faithful knock-in efficiency. Integration outcomes are influenced by the type of DNA DSBs, donor design, the use of enhanced specificity Cas9 variants, with S-phase regulated Cas9 activity. Moreover, restricting Cas9 expression with a self-cleaving system greatly improves knock-in outcomes by substantially reducing the percentage of cells with unwanted DNA integration.

**Conclusion:**

Our results highlight the need for a more stringent assessment of CRISPR–Cas9-mediated knock-in outcomes, and the importance of careful strategy design to maximise efficient and faithful transgene integration.

**Supplementary Information:**

The online version contains supplementary material available at 10.1186/s12929-024-01020-x.

## Background

The development of CRISPR–Cas technology as a genome editing tool has revolutionized the field of biology [1]. Introduction of DNA double strand breaks (DSBs) at predetermined locations by the CRISPR–Cas9 nuclease system, has enabled the generation of intended DNA changes to specific locations in the genome. The potential of changes to the targeted DNA is nothing sort of unlimited, which could include single base changes, deletions, as well as insertions, or knock-in (KI) of short and long transgene sequences.

Indeed, proof-of-concept of targeted transgene integrations using CRISPR–Cas9 has been presented in early studies with relatively low efficiencies [2–4]. Multiple strategies have been developed to increase the efficiency of targeted integration. This includes augmentation of DNA repair machinery [5–8], specific design of transgene donor template [9–17], enhancing Cas9 specificity [18–23] and reducing [24–31] or regulating Cas9 activity [32–34]. Individual strategies were reported to increase the efficiency of transgene integrations compared to the conventional CRISPR–Cas9 targeted integration with the possibility that combination of strategies could enhance the performance of integration even further. In general, homologous sequences flanking the transgene and the use of enhanced specificity Cas9 or cell cycle regulated Cas9 activity was shown to be most promising.

Despite these improvements on efficiency of transgene integrations, little attention was given to assess the faithful-ness of integration. Faithful integration is often defined as intended on-target transgene integration assessed by phenotypic (transgene expression) and genotypic (no unwanted mutation at the target junction) evaluation. However, this focus on on-target integration could lead to unwanted integration events elsewhere being missed. Indeed, there are growing reports of detectable unwanted integration events from CRISPR–Cas9 mediated gene editing [35–42]. This suggest that current CRISPR–Cas9 mediated gene integration methods are prone to unwanted events that may or may not be detectable during initial assessment [38, 40, 41]. Most CRISPR KI assays used in determining the fidelity of CRISPR editing focus on the junctions between the integrated transgene and the target loci, as well as at related off-target sites. While these sequencing strategies ensure high resolution information on the sequence level, they fail to report other unwanted events [35–37].

In order to evaluate the efficiency as well as the faithful-ness of target transgene integration events, or fidelity, we have developed a novel transgene integration assay that allows the efficient and rapid evaluation of multiple strategies in parallel. With this assay we tested multiple established KI strategies to explore the main factors that influence CRISPR–Cas9 gene editing outcomes.

## Methods

### CRISPR Cas9 expressing plasmids

All expressing plasmids were developed in Dr. Adam West laboratory. Information on the plasmid map and sequences used in this study are provided in Additional file 1.

### CRISPR sgRNA design and cloning

The AAVS1 target sequence was adapted from Mali et al. [2]. Self-cleaving guides targeting the Cas9 expressing plasmid, was designed using the CHOPCHOP tool (https://chopchop.uib.no) targeting the N-terminus of the Cas9 coding sequence. Self-targeting sequences are provided in Table 1. Oligonucleotides of the guides were purchased from IDT Technologies.

**Table 1:** Self-cleaving gRNA sequences targeting Cas9-expressing plasmids

Guide	Sequence
scA37	AGAACTTTGAATTTTTTGCT
scB93	GCGCCCTCCTGTTCGACTCC
scA74	GGTTCAGGTCCCCCTCGATG
scB97	GAGAACCCGATCAACGCATC

Oligonucleotide complement pairs were annealed by gradually decreasing the temperature from 96 to 25 °C with a − 0.3 °C/s rate between steps using a thermal cycler (Bio-rad). Annealed oligonucleotides were ligated to BbsI-digested guide expressing plasmid with DNA ligase IV (New England Biolabs) and incubated at 16 °C overnight and then transformed in DH5α *E. coli* competent cells (Agilent Technologies). Plasmids containing the guide sequences were then validated by PCR.

Multi-guide expressing plasmids were constructed by Golden Gate assembly. Briefly, pGuide containing the self-cleaving sgRNA and pMulti-empty plasmid were mixed with BsmBI/Esp3I (New England Biolabs) and T4 DNA ligase (New England Biolabs), incubated at 37 °C for 10 min, 16 °C for 15 min for 5 cycles, followed by deactivation at 37 °C for 30 min and 80 °C for 5 min. The reaction was then transformed into SURE 2 Supercompetent cells (Agilent Technologies), and the assembled plasmids were verified by colony PCR and restriction digestion.

### Cloning geminin into pMulti Cas9 expressing plasmid

330 bp Geminin fragment was amplified using Herculase Polymerase II (Agilent Technologies. Correct amplicons were then purified with a QiAquick PCR Purification kit (Qiagen). Parental pMultiCas9-mAmetrine plasmids were digested, verified, and purified before fusion cloning with the Geminin fragment. The fusion master mix was incubated and transformed into Subcloning DH5α competent cells (Agilent Technologies). Correctly assembled plasmids were verified by colony PCR and restriction digestion before isolation with Qiagen Plasmid Midi.

### AAVS1 donor plasmid cloning

495 bp of the AAVS1 locus was amplified from genomic extracts with Herculase Polymerase II (Agilent Technologies) using a Forward AAVS1AscI primer (GGA TCC GGC GCG CCC CCC GTT CTC CTG TGG ATT C) and a Reverse AAVS1XhoI primer (GGA TCC CTC GAG ATC CTC TCT GGC TCC ATC GT). Thermal cycler reaction conditions were performed with an annealing temperature of 52 °C. 10 μl of PCR product was visualized through 1% agarose gel. Samples with the correct band size were then purified with the QiAquick® PCR Purification kit.

5 μg of AAVS1 amplicon, as well as 5 μg of parental pSHTLR5 and pSHTLR3 plasmids were digested with 1 μl AscI-HF (New England Biolabs), 1 μl XhoI-HF (New England Biolabs) at 37 °C for 1 h. 1 μl of CIP (New England Biolabs) was added into the reaction with the pSHTLR5 and pSHTLR3 and incubated for a further 1 h. Linearisation of plasmids and digestion of AAVS1 amplicon were verified using a 1% agarose gel and purified with QiAquick® PCR Purification kit.

Ligation was performed using T4 DNA ligase (New England Biolabs) following manufacturers instructions. 2 μl of the ligated reaction was then transformed into DH5α competent cells (Agilent Technologies) according to the manufacturer’s protocol. Correctly assembled plasmids were then verified by colony PCR. Correct colonies were expanded, and plasmids were isolated using Qiagen Plasmid Midi (Qiagen) for Gateway cloning.

Gateway cloning utilized the LR clonase system (ThermoFisher Scientific) which assembles four plasmids together. The final donor vector contains fragments from pSHTLR5-AAVS1 and pSHTLR3-AAVS1, which contains the AAVS1 fragments, pSHTLRmid-SA-eGFP-PURO and pDEST R4-R3-CAG-BFP which contains transgene and the backbone, respectively. 10 fmol of pSHTLR5, pSHTLRmid, and pSHTLR3 were mixed with 20 fmol of the backbone vector, pDEST R4-R3-CAG-BFP, in TE buffer pH 8.0. The reaction was performed using LR clonase II Plus enzyme mixture (Invitrogen) following the manufacturers instruction. 2 μl of the reaction were transformed into DH5α competent cells (Agilent Technologies) according to the manufacturer’s instruction. Correctly assembled plasmids were then verified by colony PCR and restriction digestion with BamHI (New England Biolabs) and HindIII (New England Biolabs). Correct colonies were expanded, and plasmids were isolated using Qiagen Plasmid Midi (Qiagen).

### Cell culture and transfections

HEK293T were cultured in Dulbeco’s modified Eagle’s medium (DMEM) (Thermo Fisher Scientific) supplemented with 10% Foetal Bovine Serum (FBS) (Thermo Fisher Scientific) and 100 U/ml penicillin/streptomycin (Gibco). Cells were maintained in 37 °C and 5% CO_2_ in a humidified incubator. Cells were passaged every 2 days or until reaching 80% confluency.

Cell transfections were performed using Lipofectamine 3000 (Thermo Fisher Scientific) according to the manufacturer’s protocol with a molar ratio of DNA:lipofectamine of 4:3. 3 × 10^5^ cells were seeded 24 h before transfection to achieve a 60% confluency upon transfection. A total of 3 μg of Cas9 plasmid, 3 μg of donor plasmid, and 3 μg of guide RNA plasmid were transfected.

K562 cell lines were cultured in Roswell Park Memorial Institute (RPMI) medium supplemented with 10% FBS and 100 U/ml penicillin/streptomycin. Cells were maintained in 37 °C and 5% CO_2_ in a humidified incubator. Cells were passaged every 2 days or until reaching a concentration of 8 × 10^5^ cells/ml.

K562 cells were transfected by nucleofection with the 4D-Nucleofector™ system (Lonza). 2 × 10^5^ cells were transfected with the FF-120 program following suggested manufacturers manual. A total of 3 μg of Cas9 plasmid, 3 μg of donor plasmid, and 3 μg of guide RNA plasmid were transfected.

### Genetic integration assay

Flow cytometry was performed 2 days after transfection to evaluate transgene integration and transfection efficiency. To enrich for transgene integration, 1 μg/μl Puromycin (Thermo Fisher Scientific) was added to the medium 3 days after transfection. Cells were kept growing in puromycin selected medium for 21 days prior to collection. Cells were collected and washed with DPBS by centrifugation at 100×*g* for 10 min. Cell pellets were resuspended in 200 μl of medium and analysed using the Attune NxT Acoustic Flow Cytometer (Thermo Fisher Scientific).

### DNA repair inhibitor preparation and storage

DNA repair inhibitors of NU7441, Rucaparib, and B02 were purchased as lyophilised powder (Merck). Pellets were spun down before diluting with Dimethyl sulfoxide (DMSO) (Sigma-Aldrich). Working stock were then diluted and filtered before application. Both stock and working stock are stored in − 80 °C freezer.

For DNA repair inhibition experiments, cell cultures were treated with DNA repair inhibitors 1 h prior transfections. Concentrations used for CRISPR–Cas9 KI experiments are 2 µM for NU7441, 10 µM for Rucaparib, and 10 µM for BO2. Plasmid transfections, puromycin selection, and cellular evaluations by flow cytometry were performed described above.

### Western blot

HEK293T cells were transfected with a Cas9 expressing plasmid by Lipofectamine 3000. Cells were collected and analyzed by flow cytometry 2 days after transfection. Nuclear extracts were prepared and quantified by Bradford assay.

To evaluate the self-targeting system, HEK293T cells were transfected with Cas9-expressing plasmids containing Cas9-self-cleaving gRNA sequences. Cells were collected and analysed by flow cytometry 2 days after transfection. Nuclear extracts were prepared and quantified by Bradford assay. 15 μg samples were mixed with a reducing agent and the loading buffer was then incubated in 75 °C for 15 min. Samples were allowed to cool down in room temperature before running it in a NovexTM 4–12% Tris-Glycine Base gel (Invitrogen). Nitrocellulose transfer was done by using the Bolt tank (Thermo Fisher Scientific) with the High MW settings. The membranes were blocked with TBST containing 5% low fat milk for 1 h. Membranes was washed with TBST four times before incubation with 1:2000 of Cas9 monoclonal antibody (Diagenode) or TBP antibody (SantaCruz) overnight in 4 °C with mild shaking. On the next day, membranes were washed with TBST four times and incubated with 1:2000 secondary anti-mouse antibodies (SantaCruz) for 2 h. Antibody detection was done by adding West Femto substrate (Thermo Fisher Scientific) to the membrane and detected with the Biorad Gel-doc.

### Statistical analysis

Statistical analysis was performed in Excel using unpaired two sample student t-test, one way-Anova, and post-hoc Tukey test. A p value of < 0.05 was considered as statistically significant.

## Results

### Development of a CRISPR–Cas9 transgene assay to evaluate knock-in fidelity

We have developed a novel fluorescence-based transgene integration assay that allows the quantification of integration events involving both the transgene and the plasmid backbone. This assay provides a platform for the subsequent testing and development of improvements to knock-in strategies.

**Fig. 1:**
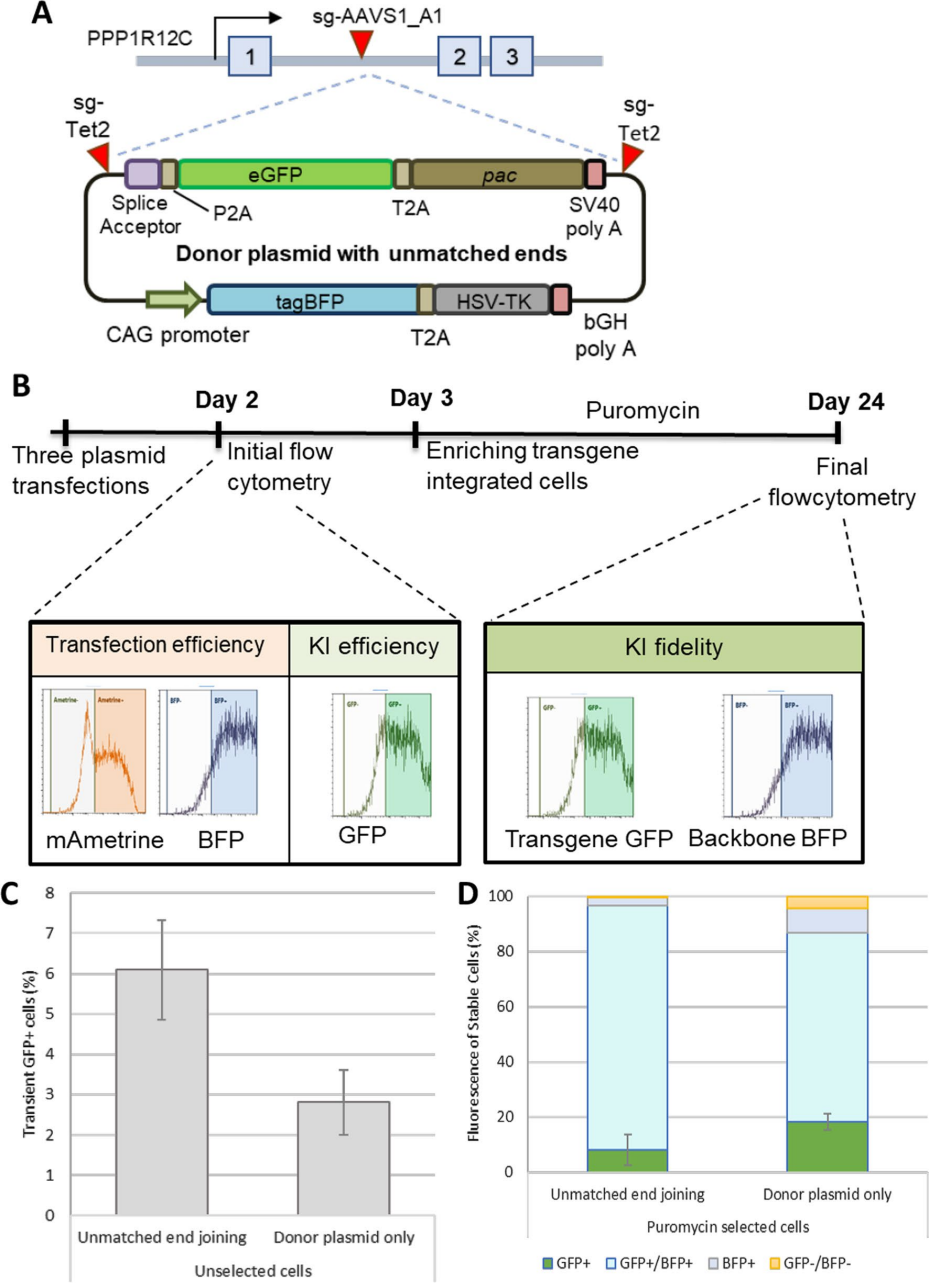
Development of the CRISPR–Cas9 knock-in assay. **A** Donor plasmid design for unmatched end joining strategies. The AAVS1 locus is targeted by CRISPR–Cas9 using the AAVS1-A1 guide. Cas9-targeted cleavage will form DNA DSBs at which the transgene is to be inserted. The unmatched end donor plasmid contains the transgene, which is a splice acceptor—driven EGFP coupled with the *pac* puromycin resistance gene for further selection. On the backbone, BFP expression is driven by a CAG promoter so as to report backbone integration. The donor plasmid contains Tet2 guide cleavage sites on either side of the transgene but does not have any sequences that are homologous to the AAVS integration site. **B** Overview of the knock-in assay. Three plasmids are transfected into HEK293 cells (i) Cas9 (wt or mutant) and mAmetrine expression and (ii) expression of sgRNAs for AAVS1 and Tet2, (iii) the unmatched end donor plasmid. Initial assessment by flow cytometry quantifies mAmetrine and BFP expression for transfection efficiency, and GFP to evaluate knock-in efficiency. Puromycin selection is then applied to enrich for cells in which the transgene is integrated and expressed. Final flow cytometry assessment is performed to collect GFP and BFP expression data to evaluate knock-in fidelity. **C**, **D** Data from the knock-in assay using wtCas9 and the unmatched end donor plasmid. **C** Flow cytometry quantification of GFP expressing cells 2 days after transfection. The donor plasmid only control was not transfected with the Cas9 and sgRNA plasmids. **D** Fluorescence profile of puromycin resistant cells after transfection and selection according to the unmatched end joining knock-in strategy. Error bars in **C** and **D** represent the standard error mean from three independent replicates. Students’ T tests indicate no significant differences between the donor only control and the unmatched end joining experiment. p > 0.05

The assay is plasmid-based, as this allows the flexibility to rapidly test different strategies by using available plasmid vectors for Cas9, guide RNAs and transgene donors (Fig. 1). The Cas9 expressing plasmid contains a CMV-driven Cas9 coding sequence followed by a T2A self-cleavage peptide and the mAmetrine reporter gene. The T2A sequence encodes a ‘self-cleaving’ amino sequence, adapted from an insect virus, *Thosea asigna* [43]. These 2A peptide sequences have been widely used for co-expressing multiple proteins from a single mRNA. The mechanism of ‘cleavage’ involves ribosome skipping at the C-terminus of the 2A producing two separate proteins. This design enables the evaluation of transfection efficiency, where co-expression of mAmetrine would be a marker for in vivo Cas9 expression.

To ensure stable expression of integration we selected a guide targeting the intron 1 of the adeno-associated virus locus 1 (AAVS1) genomic safe harbour which is often used as a target for CRISPR–Cas9 editing site [2] (Fig. 1A).

Evaluation of transgene integration was done by assessing the integration of donor fragments containing a GFP gene (Fig. 1A). The donor plasmid contains the transgene, which encodes a splice acceptor (SA)-driven EGFP followed by a T2A peptide and the *pac* puromycin resistance gene for antibiotic selection. This promoter-less reporter cassette ensures the transgene is only expressed if integrated in the correct location at the correct orientation. On the backbone of the plasmid, a CAG promoter-driven BFP fluorescence gene is used to report transfection efficiency and unwanted exogenous DNA integration. Cas9-mediated linearization of the donor plasmid can be induced using sgRNAs targeted to either end of the transgene.

To perform the assay, HEK293T cells are transfected with three plasmids; Cas9 expressing plasmid, sgRNA expressing plasmid, and the donor plasmid (Fig. 1B). Two days after transfection, cells are analysed by flow cytometry to evaluate transfection efficiency using plasmid-driven mAmetrine and BFP expression. The efficiency of initial transgene integration is assayed using GFP expression.

Cells were then selected with puromycin, to allow time for the transient, plasmid-based BFP expression to be lost while maintaining cells in which the GFP transgene has integrated. Subsequent flow cytometry analysis of the selected cells was used to quantify any unwanted integration of the donor plasmid by detecting BFP expression while faithful transgene integration measured by GFP expression does not contain BFP (Additional file 1: Fig. S1).

In the unmatched ends knock-in approach, Cas9 cleavage is directed at the AAVS1 locus and at either ends of the transgene in the donor plasmid, generating two plasmid DNA fragments. There is no sequence homology between the donor plasmid and the integration site. Initial transgene integration efficiency was 6.1% as indicated by GFP expression 2 days after transfection (Fig. 1C). Puromycin selection resulted in cells that were mainly GFP+BFP+ (Fig. 1D), and quantification revealed that 88.6% of the puromycin-resistant GFP expressing cells also express BFP. Only 8% of the puromycin enriched cells are GFP+BFP− (Fig. 1E). This knock-in profile of CRISPR–Cas9 edited cells was not significantly different compared to the donor plasmid control that did not include the CRISPR system.

Additionally, the data reveal that puromycin-resistant cell cultures are a polyclonal mix that are dominantly composed of cells that co-express GFP and BFP. This provides strong evidence that integration of unwanted exogenous DNA is highly pervasive in edited cells.

### High occurrence of backbone integration reduces the fidelity of CRISPR–Cas9 mediated transgene integration

**Fig. 2:**
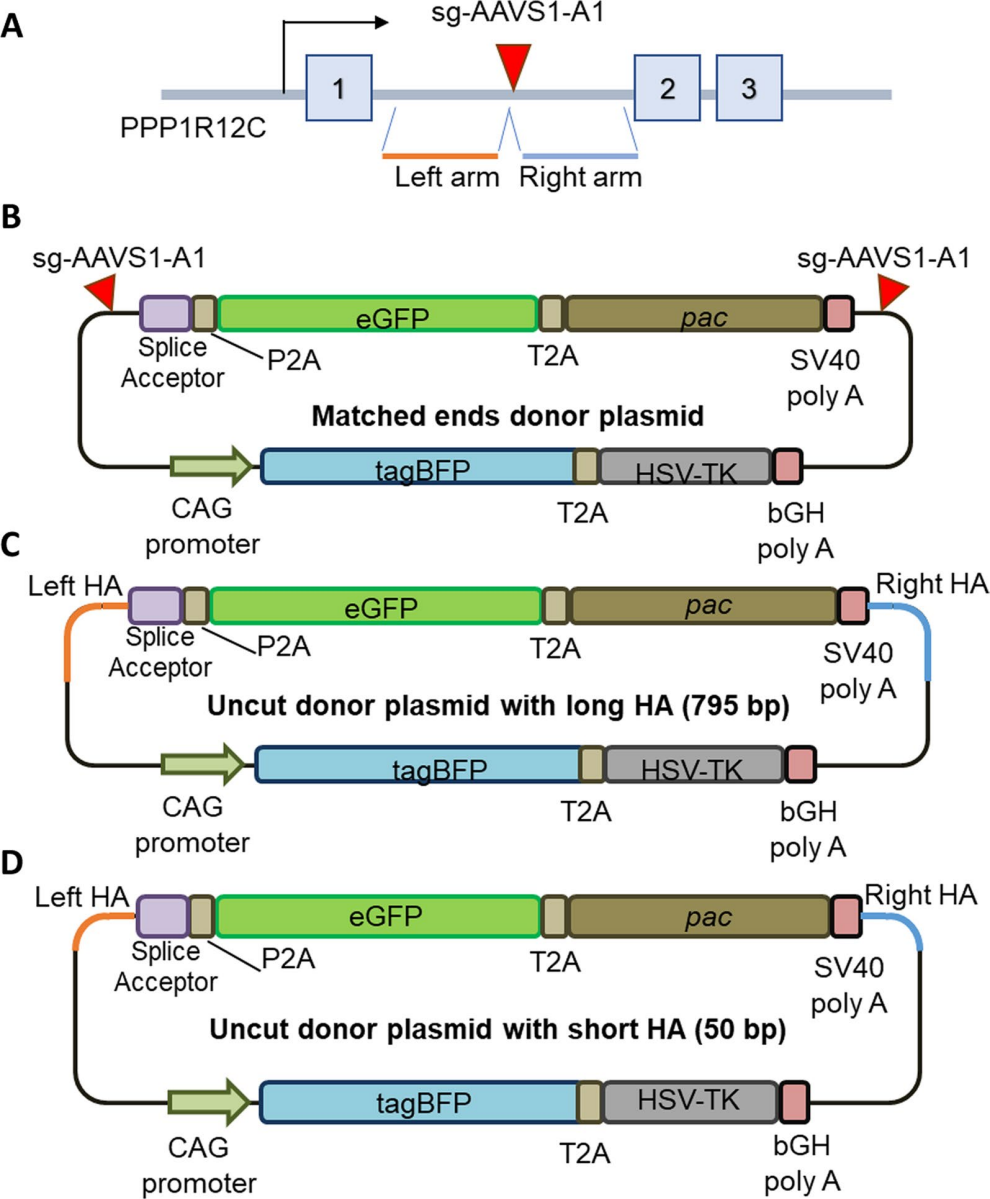
Donor plasmid design for matched end joining and HDR-based strategies. **A** AAVS1 locus is targeted by CRISPR–Cas9 using AAVS1-A1 guide. Cas9-targeted cleavage will create DNA DSBs where the transgene of interest is intended to be inserted. The left and right arms either side of the DSB may be incorporated into the donor template to direct on-target integration (see **C** and **D**). **B**–**D** The donor plasmids contain a splice acceptor—driven GFP transgene coupled with the puromycin resistance gene, and BFP driven by a CAG promoter on the plasmid backbone. **B** For the matched end joining strategy, the donor plasmid contains a copy of the AAVS1 sgRNA target site at both flanks of the transgene. Cas9 cleavage in vivo using the AAVS1-A1 guide would cleave the endogenous locus while also generating two DNA fragments from the plasmid, the transgene and the backbone. **C**, **D** For homology-directed repair strategies, the transgene is flanked by sequences corresponding to the left and right arms of the endogenous locus. However, the donor plasmids have a mutated PAM sequence of the AAVS1-A1 sequence target, preventing Cas9 cleavage. This ensures the HR-based plasmids remain circular to initiate the HR mechanism. Homology arms are 795 bp and 50 bp in the long and short HA donor plasmids, respectively (**C**, **D**). Orange lines indicate left arm homology. Blue lines indicate right arm homology

Our initial evaluation of CRISPR–Cas9 strategy involved the linearization of donor plasmid without any homologous sequences to guide on-targeted integration (Fig. 1). Various strategies have previously been developed to increase the efficiency of targeted integrations [5–34]. We adopted these strategies and evaluated their performances with our assay (Fig. 2).

The “matched ends” strategy still involves the cleavage of the donor fragment at either side of the transgene, with 200 bp of sequence that is homologous to the AAVS1 target included on either side of the transgene fragment (Fig. 2B).

Two other donor plasmids were designed to explore homology directed repair (HR)-based pathways (Fig. 2C, D). Some reports have shown that the length of homology sequences at both ends of the transgene (from herein referred to as homology arms or HA) determines the efficiency of integration [11, 15, 16]. To evaluate the impact of the length of homology arms in our KI assay, we designed short (50 bp) or long (795 bp) HAs flanking the target site, to be referred to as short HA and long HA, respectively. To ensure that the donor plasmids are processed with HR machinery and not non-homologous end joining (NHEJ) or microhomology-mediated end joining (MMEJ), we modified the targeting sequence on the homology arm by deleting the PAM sequence of the CRISPR target site ensuring no Cas9 targeted cleavage on the donor plasmid. This modification will ensure the donor plasmids remain circular and must rely on strand invasion for on-target.

**Fig. 3:**
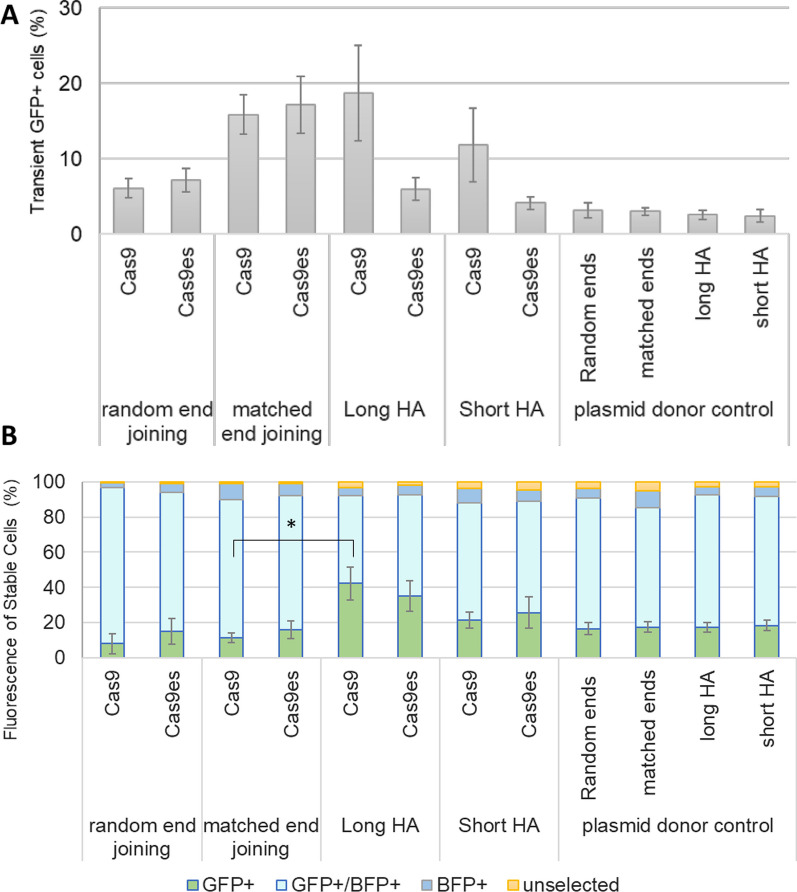
High occurrence of backbone integration in all CRISPR–Cas9 mediated KI strategies. **A** Quantification of GFP expressing cells 2 days after transfection. GFP can only be expressed when the donor plasmid is integrated into the genome downstream of a transcribed RNA splice donor site. HEK293T cells were transfected with Cas9 and sgRNA plasmids along with the donor plasmid for either the unmatched ends, matched ends, long HA, or short HA strategy. Plasmid donor only transfections were performed as a control for random plasmid integration into the genome. Error bars indicate standard error from 3 biological replicates. **B** Fluorescence profile of puromycin-selected cells. Transfected cells were selected with puromycin for 3 weeks prior to Flow cytometry analysis. Any BFP expression arising from the backbone of the transiently transfected plasmid is lost by this time, so the relative proportions of cells expressing GFP and/or BFP can be calculated. The percentages of green, blue, and double fluorescent cells are plotted as stacked graphs. Error bars indicates standard error for GFP+ cells from three biological replicates. *p < 0.05 in student T-test

The initial transgene integration efficiencies of each strategy indicate that the matched end joining and long homology arms strategies both had more efficient transgene integration than the unmatched end joining strategy and donor control, although these differences did not reach statistical significance (Fig. 3A).

Interestingly, flow cytometry of selected cells produced different outcomes (Fig. 3B). The KI profile of the matched end joining strategy was similar to the unmatched end joining strategy. However, the long HA strategy generated a significant increase in KI fidelity compared to unmatched and matched end joining strategies (p = 0.05 and p = 0.03, respectively).

Our assay system also allows the direct comparison of different Cas9 variants. We compared wild type SpCas9 with espCas9 (1.1), a rationally engineered variant of Cas9 that has reduced off-target cleavage [21]. However, the use of espCas9 (1.1), from herein referred to Cas9es, did not significantly alter the KI fidelity of any of the strategies tested.

This data indicates that use of a homology-directed repair strategy for inserting transgenes leads to a significant reduction in unwanted integration of exogenous DNA.

### The type of DNA DSBs influence KI outcomes

**Fig. 4:**
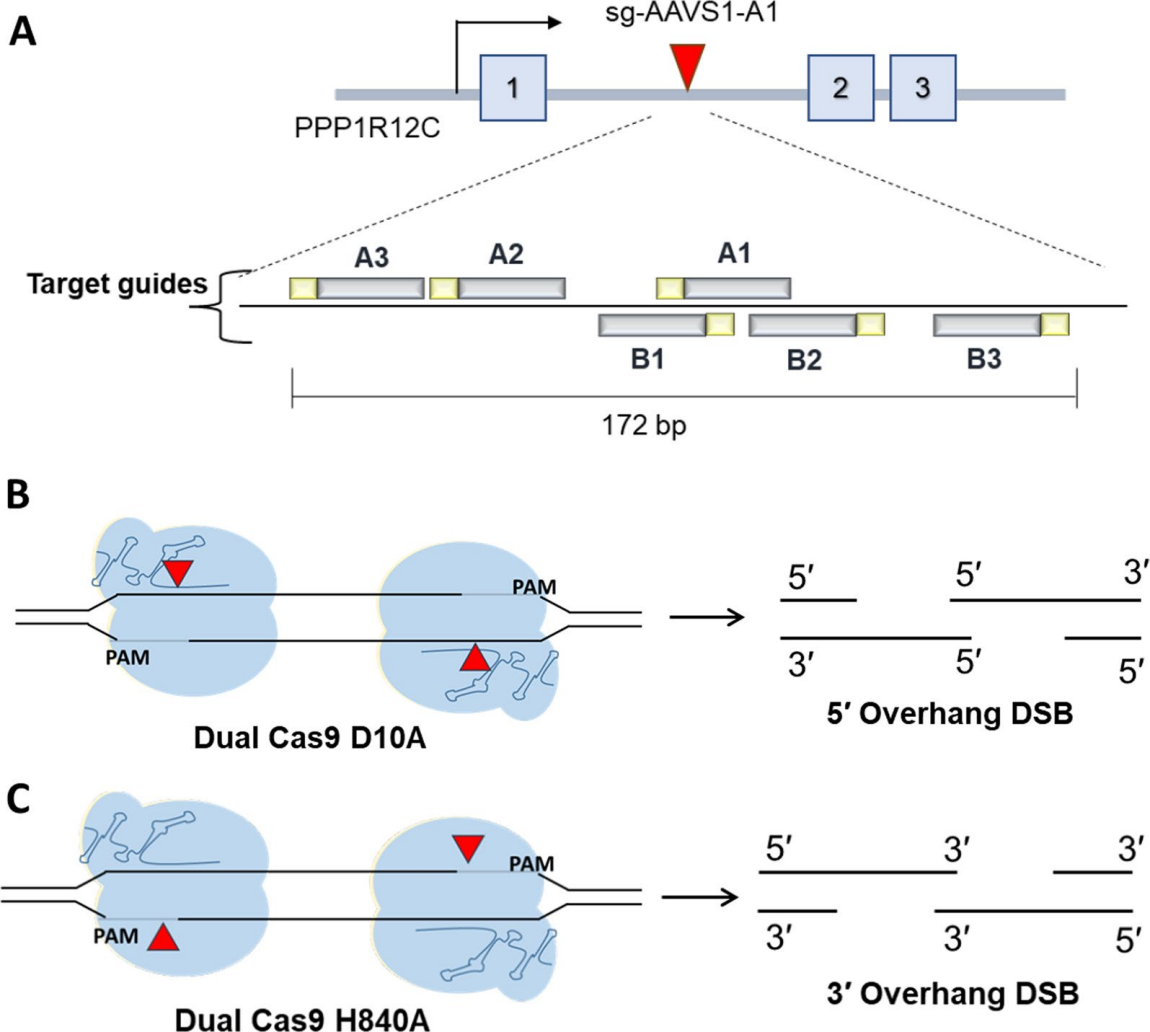
Dual Cas9 nickase strategy mediates DNA DSBs with single stranded overhangs. **A** 6 different guides were used for the dual Cas9 nickase strategy. Three guides are complement to the sense strand (Guide A1, A2, and A3) and three guides target the anti-sense strand (Guide B1, B2, and B3). Two different Cas9 nickases were applied, Cas9 D10A and Cas9 H840A. **B** Two Cas9 D10A utilizing guides at a tail-to-tail orientation, will generate a DNA DSB with 5ʹ overhang ssDNA. As for **C** dual Cas9 H840A strategy will generate a 3ʹ overhang DNA DSB

We next evaluate whether the type of CRISPR–Cas9 mediated DNA DSBs would induce distinct integration outcomes. Targeting the AAVS1 locus, we adopted six guides complement to the sense and antisense strand of the target locus [2] (Fig. 4A). Targeting guides are paired with a tail-to-tail orientation, as upon utilizing paired Cas9 D10A and H840A variants will generate 5ʹ or 3ʹ overhangs single stranded DNA (ssDNA) overhangs, respectively (Fig. 4B, C). As it has been reported that the length between Cas9 binding site and the orientation of the PAM is critical for efficient targeting [2, 4, 44, 45] we paired these AAVS1 guides to generate DNA DSBs with various lengths of overhangs (Additional file 1: Table S1). The different lengths of overhangs would enable us to evaluate the impact of ssDNA homologous sequences on transgene integration.

**Fig. 5:**
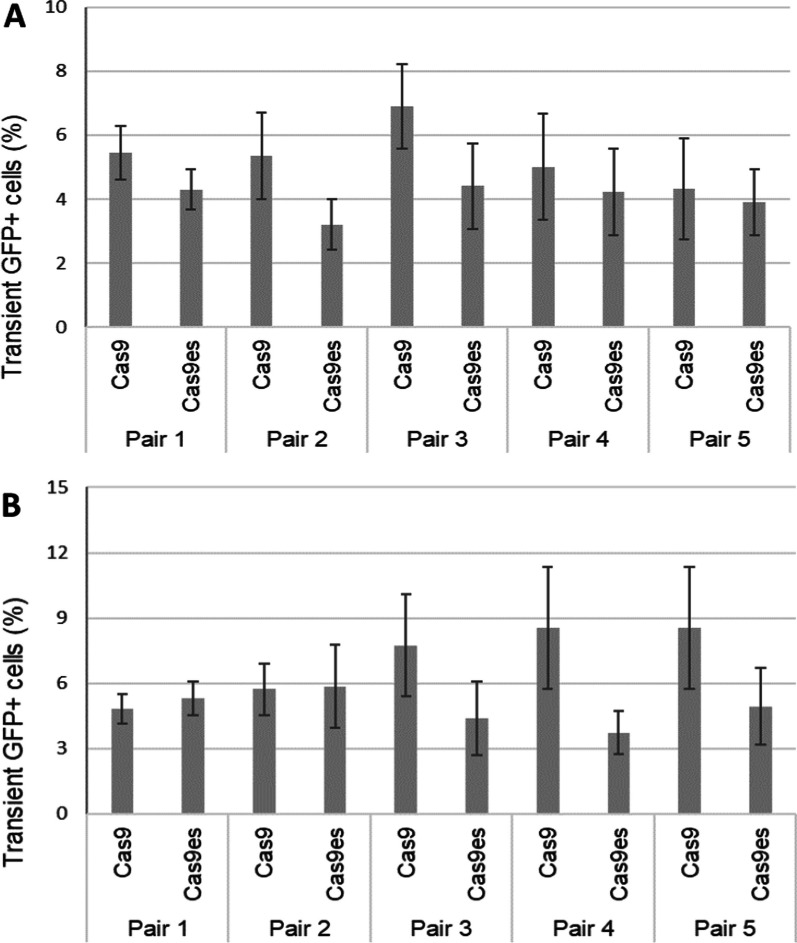
No significant differences in the efficiency of transgene integration between different lengths of overhangs. HEK293T cells were transfected with plasmids expressing either Cas9 D10A nickases or H840A nickases, alongside plasmid containing AAVS1 pair guides and the donor plasmid. Cells were analysed for GFP expression by flow cytometry 2 days after transfections. **A** Strategies utilizing 5ʹ overhang DNA DSBs with various lengths of overhangs; **B** strategies utilizing 5ʹ overhang DNA DSBs with various lengths of overhangs. Error bars indicate standard error from three biological replicates. *p < 0.005

**Fig. 6:**
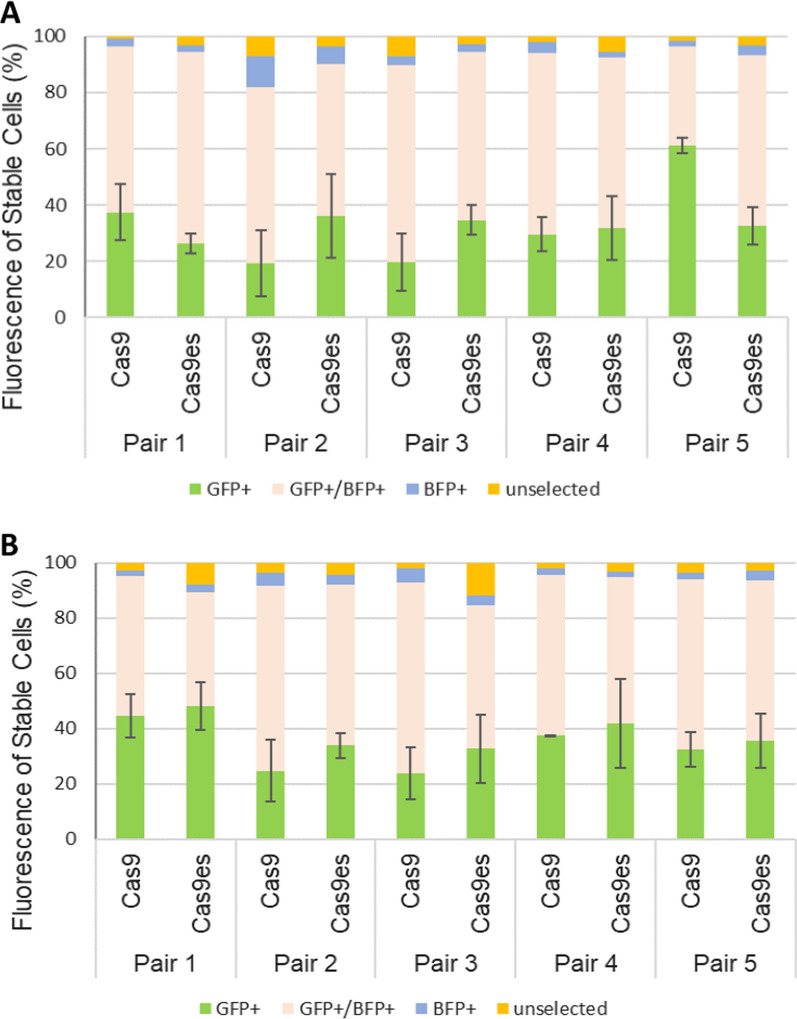
Distinct integration profiles of stable puromycin selected cells from overhang mediated KI strategies. Transfected cells were grown under puromycin selection for 3 weeks before final flow cytometry analysis. Stable GFP+BFP− cells were quantified using flow cytometry and compared to non-Geminin results. **A** Strategies utilizing 5′ overhang DNA DSBs with various lengths of overhangs; **B** Strategies utilizing 5′ overhang DNA DSBs with various lengths of overhangs. Error bars indicates standard error from ≥ three biological replicates. *p < 0.05

Interestingly, efficiency of transgene integration was not affected by the type of DNA DSBs, the length of overhangs, and the use of enhanced specificity Cas9 variants (Fig. 5). Evaluation of stable puromycin selected cells however showed distinct integration outcome profiles for each strategy (Fig. 6). Furthermore, statistical analysis revealed that revealed that long 5ʹ overhangs, induced by dual Cas9 D10A using pair 5 guides, produced a statistically significant outcome compared to other pairs (Fig. 6A). Significant difference was also seen when comparing Cas9 and Cas9es version with pair 5 (two-sample t-test p = 0.028) indicating higher editing efficiency using the normal Cas9 D10A variant. However, no differences were observed among strategies employing 3ʹ overhang intermediates (Fig. 6B).

### The combination between Cas9es and geminin fusion increases faithful transgene integrations in long and short HA strategies

We hypothesised that restricting the formation of DSBs to S phase might have a positive effect on knock-in efficiency and fidelity, as error prone insertion through NHEJ would be minimised and HR would be promoted. Indeed, cell synchronization studies have revealed an increase of faithful editing in S-phase cells [7, 46]. Previously, two independent labs reported the development of Cas9-Geminin fusion, geminin being a DNA replication licensing protein, to enhance HR-mediated gene editing [32, 33].

To investigate whether restricting Cas9 expression to later phases of the cell cycle increases knock-in fidelity, we fused a 110 amino acid fragment of geminin to the C terminus of Cas9. We then applied this fused Cas9-geminin variant with our various KI strategies.

**Fig. 7:**
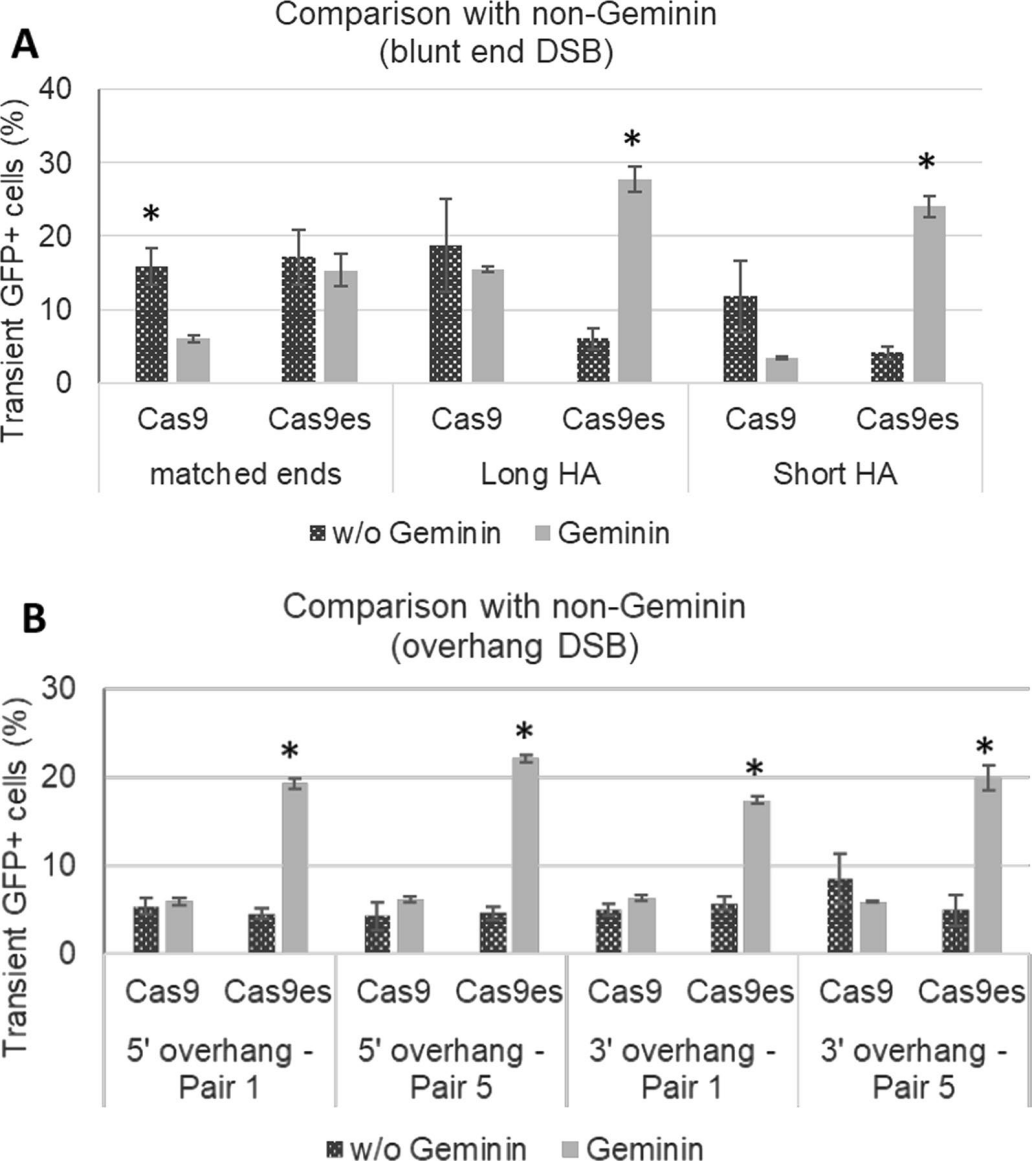
Geminin-restricted Cas9es expression increases effective transgene integration in overhang DSB-mediated strategies. HEK293T cells were transfected with plasmids expressing either Cas9, Cas9es, Cas9-geminin or Cas9es-geminin, alongside the sgAAVS1-A1 plasmid and the donor plasmid for either the matched ends, long HA, or short HA strategy. Cells were analysed for GFP expression by flow cytometry 2 days after transfections. **A** Strategies utilizing blunt end DNA DSBs; **B** strategies utilizing overhang DNA DSBs. Error bars indicate standard error from three biological replicates. *p < 0.005

Surprisingly, the geminin fusion to Cas9 directed a significant increase in initial transgene integration strategies when using the enhanced specificity Cas9es with the blunt end HR strategies (Fig. 7A). The geminin fusion led to a 4.6-fold increase in GFP+ cells with the long HA strategy and 5.8-fold increase in the short HA strategy (p < 0.005). The percentage of GFP+BFP− cells after selection was 77.7%, which is substantially higher than in previous strategies (p < 0.005).

**Fig. 8:**
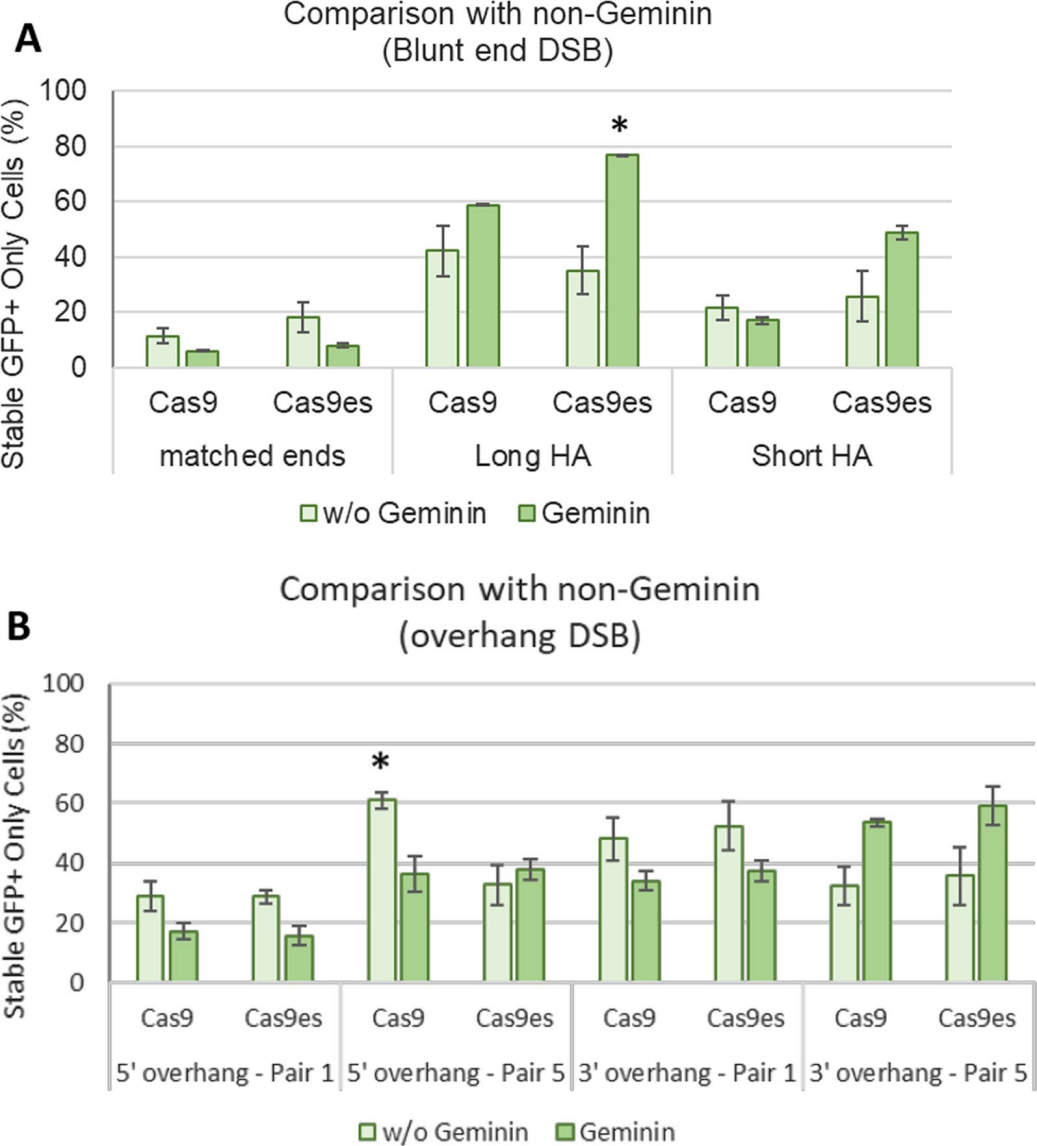
Geminin-restricted Cas9es expression increases faithful transgene integration in HA strategies. Transfected cells were grown under puromycin selection for 3 weeks before final flow cytometry analysis. Stable GFP+BFP− cells were quantified using flow cytometry and compared to non-Geminin results. **A** Strategies utilizing blunt end DNA DSBs; **B** strategies utilizing overhang DNA DSBs. Error bars indicates standard error from ≥ three biological replicates. *p < 0.05

The effect on geminin on Cas9es experiments was also substantial in both 5ʹ and 3ʹ overhang strategies (Fig. 7B). Both short and long 5ʹ overhang DSB intermediates displayed a significant increase of transient GFP+ cells upon using the Cas9es-geminin fusion (p < 0.01). However, this fusion combination produces lower selected GFP+BFP− cells compared to blunt end strategy (Fig. 8). Integration fidelity was not affected by the length of overhang nor the use of enhanced specificity Cas9 variant. Taken together, the data suggest that strategies using overhang DSB intermediates are repaired and integrated more rapidly in the S phase of the cell cycle, but that faithful integration is independent of the timing of the DSB formation.

### Reducing Cas9 expression increase faithful transgene integration

Reducing Cas9 activity has been shown to reduce off-target activity [47, 48]. Various inducible expression systems have been developed to restrict Cas9 expression [30, 49, 50]. However, these approaches suffer from decreased targeting activity and the requirement for an induction step. One alternative strategy that would alleviate these disadvantages is to apply a self-cleaving system [24, 28].

The concept of the CRISPR Cas9 self-cleaving system is to introduce a guide RNA that targets the coding sequence of the expressing vector. Cas9 targeting and cleavage of the expressing vector initiates DNA degradation and limits Cas9 expression, thus reducing unwanted off-target activity [28, 31].

**Fig. 9:**
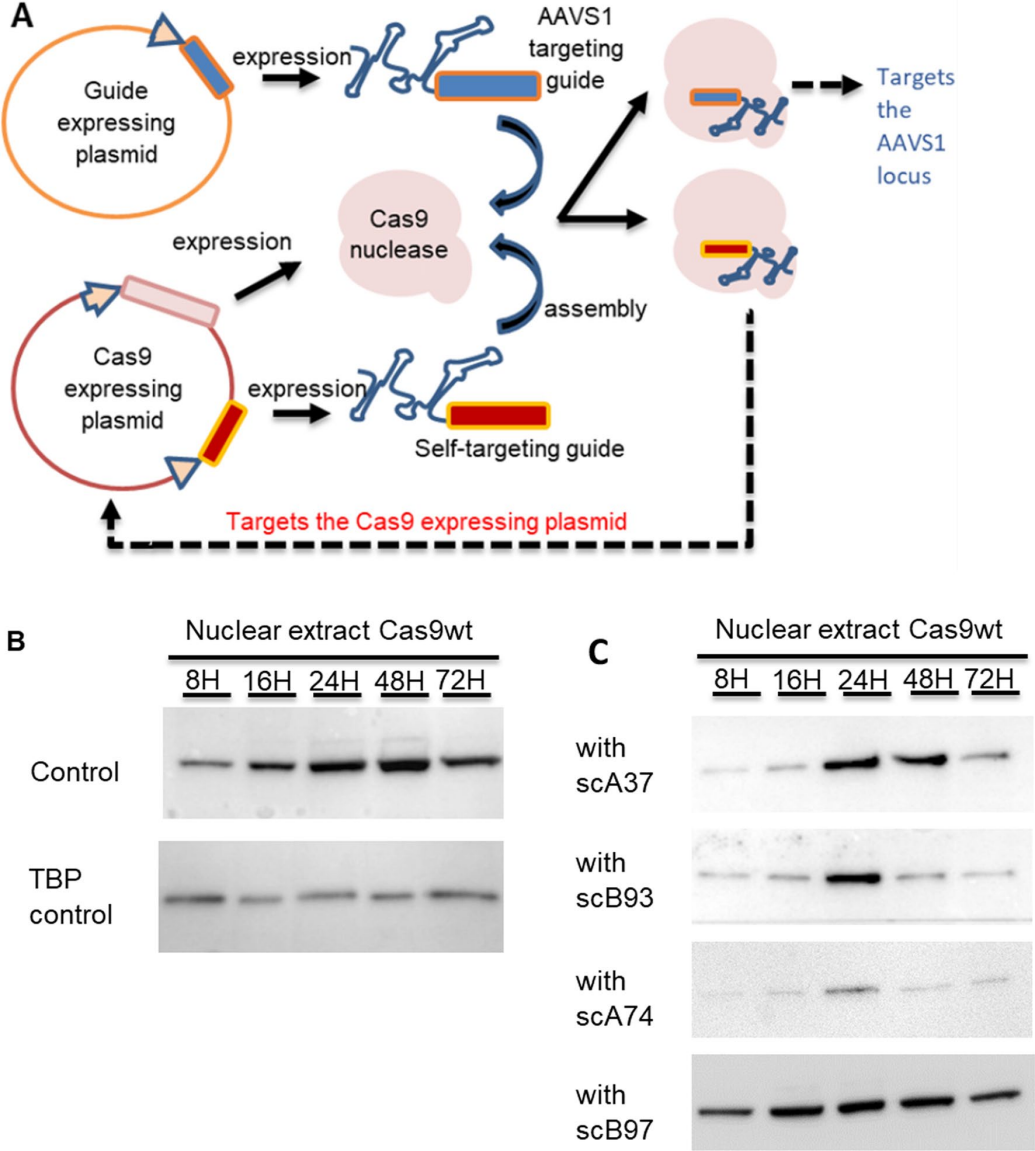
The self-cleaving Cas9 system. **A** The plasmid based self-limiting system incorporates a self-targeting guide into the Cas9 expressing plasmids. Upon expression, Cas9 assembly will generate two Cas9 complexes; one targeting and cleaving the AAVS1 genomic locus and the other cleaving the Cas9 expressing plasmid. **B**, **C** HEK293T cells were transfected with Cas9 expressing plasmids with their appropriate self-targeting guides. Cells were collected at each time points (8, 16, 24, 48, and 72 h after transfection) and nuclear extracts were obtained. Western blotting was used to detect Cas9 protein (161 kDa) or the TBP loading control (37.6 kDa) in **B** control and **C** cells transfected with Cas9 self-cleaving guides

The benefits of reduced off-targeting activity have been demonstrated in genetic knock-out (KO) purposes. We hypothesized that reduction in Cas9 expression would also aid CRISPR–Cas9 mediated KI strategies. We, therefore applied a self-limiting system to our assay to evaluate the effects on KI efficiency and fidelity (Fig. 9A).

Four self-cleaving guides targeting the N terminus of the Cas9 coding sequence were evaluated for their ability to restrict Cas9 expression following plasmid transfection. All self-cleaving guides reduced Cas9 protein levels during the 72-h evaluation (Fig. 9B). Self-cleaving guide A74 generated the greatest reduction in Cas9 expression and was used in subsequent experiments.

The use of the self-cleaving system significantly increased transgene integration efficiency in all the strategies tested (Table 2). Strikingly, an increase in KI fidelity was also seen in almost all the strategies, with all the long HA strategies achieving over 78% GFP+BFP− cells.

**Table 2:** Overall strategies that produced high intended transgene integration outcomes

Outcome	Strategy	Cas9 variant	Transient GFP+ (%)	Selected GFP+ (%)	Consensus conclusion
High efficiency high fidelity	Long HA	Cas9-SC	65.4	81.1	Long HA with SC system
	Long HA	Cas9-Gem-SC	66.5	80.7	
	Long HA	Cas9es-Gem-SC	64.4	78.9	
	Long HA	Cas9es-SC	68.8	78.3	
High efficiency low fidelity	Short HA	Cas9-SC	64.9	29.8	Blunt end-mediated KI strategies with SC system
	Short HA	Cas9es-Gem-SC	61.5	32.8	
	Short HA	Cas9-Gem-SC	60.8	38.4	
	Matched end joining	Cas9-Gem-SC	60.0	27.6	
	Matched end joining	Cas9es-SC	57.3	18.7	
	Short HA	Cas9es-SC	57.0	39.5	
	Matched end joining	Cas9es-Gem-SC	55.3	33.9	
	Matched end joining	Cas9-SC	54.5	14.4	
Low efficiency high fidelity	Long HA	Cas9es-Gem	27.7	77.0	Majority overhang DSB mediated KI strategies with Geminin (lesser extent SC) and long HA non-SC
	5ʹ overhang-Pair1	Cas9D10Aes-Gem	20.3	63.4	
	3ʹ overhang-Pair5	Cas9H840A-Gem-SC	2.3	62.4	
	5ʹ overhang-Pair5	Cas9D10A	4.3	61.1	
	3ʹ overhang-Pair5	Cas9H840Aes-Gem	19.9	59.4	
	long HA	Cas9-Gem	15.5	58.7	
	5ʹ overhang-Pair1	Cas9D10Aes-SC	37.2	56.5	
	3ʹ overhang-Pair5	Cas9H840Aes-Gem-SC	1.3	53.6	
	3ʹ overhang-Pair5	Cas9H840A-Gem	5.9	53.5	
	3ʹ overhang-Pair1	Cas9H840Aes	5.7	52.4	

**Fig. 10:**
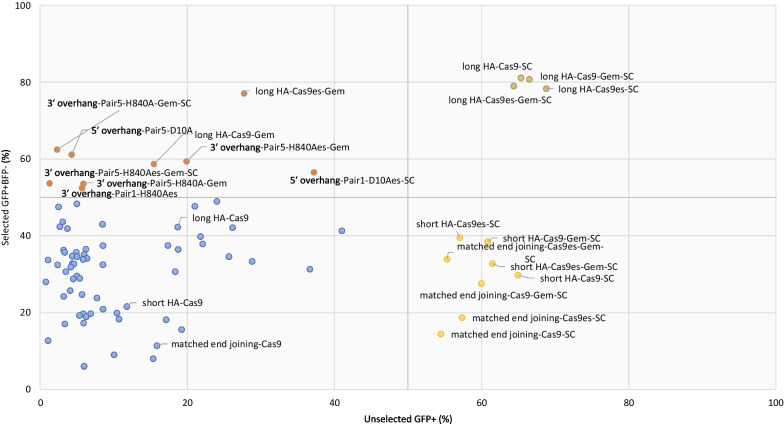
Self-cleaving Cas9 leads to substantial increases in knock-in fidelity with the long homology arm strategy. Each strategy was plotted into the dot graph for unselected GFP+ and selected GFP+BFP− results. The graph is divided into four quadrants at the 50% scale on each axis. Grouped samples are generated from the same strategy which consist of Cas9, Cas9es, Cas9-Gem, and Cas9es-Gem variants. Colour code was appointed to indicate high efficiency and fidelity (green), high efficiency with low fidelity (yellow), low efficiency with high fidelity (red), and low efficiency and fidelity outcomes (blue)

To have an overview of the performance of all tested strategies in HEK293T cells, we plotted the average GFP+ expression to an efficiency-to-fidelity plot (Fig. 10). Four quadrants were determined with the 50% of either axis generating four outcome categories: (i) high efficiency and fidelity, (ii) high efficiency with low fidelity, (iii) low efficiency with high fidelity, and (iv) low efficiency and fidelity (Table 2).

### Use of DNA inhibitory molecules leads to small but significant increases in faithful integration of short HA donors

Integration of exogenous DNA can be driven by several cellular DNA repair pathways, including NHEJ, MMEJ and HR. NHEJ is active throughout the cell cycle but is error prone. HR relies on homologous sequences in the transgene and the integration site is only active at later stages of the cell cycle. In order to investigate whether selective inhibition of different repair pathways can improve the fidelity of transgene integration, we tested three DNA repair inhibitors in our assay; NU7441 and BO2 which inhibits DNA-PK and Rad51 respectively.

DNA-PK, a member of the PI3 kinase-like kinase (PIKK) family, is recruited to a DSB end by Ku proteins [51]. Autophosphorylation on Ser2056, results in an activated form of DNA-PK which will mediate recruitment of NHEJ activator proteins such as Ligase and Artemis. NU7441 (2-*N*-morpholino-8-dibenzothiophenyl-chromen-4-one) inhibits autophosphorylation of DNA-PK resulting in an impaired NHEJ repair pathway [52, 53]. In genomic editing studies, NU7441 treatment increased HR events and reduced small indel formations [5, 6, 8].

The HR pathway is divided into Rad51-dependent repair or Rad51-independent repair pathways. Classical HR which requires strand invasion of homologous dsDNA is facilitated by Rad51 nucleofilament, promoting homologous search and strand invasion of dsDNA template [54]. Rad52 facilitates the Rad51-independent repair pathway, directing single strand annealing of homologous sequences.

B02 is a small inhibitor that inhibits Rad51 binding to the DNA and the subsequent formation of nucleofilament [55]. In CRISPR–Cas9 genetic editing experiment, B02 treatment reduces HR outcomes from single stranded oligonucleotide donor template [8]. There have yet to be any reports on the effect of Rad51 in HR processing of dsDNA templates.

**Fig. 11:**
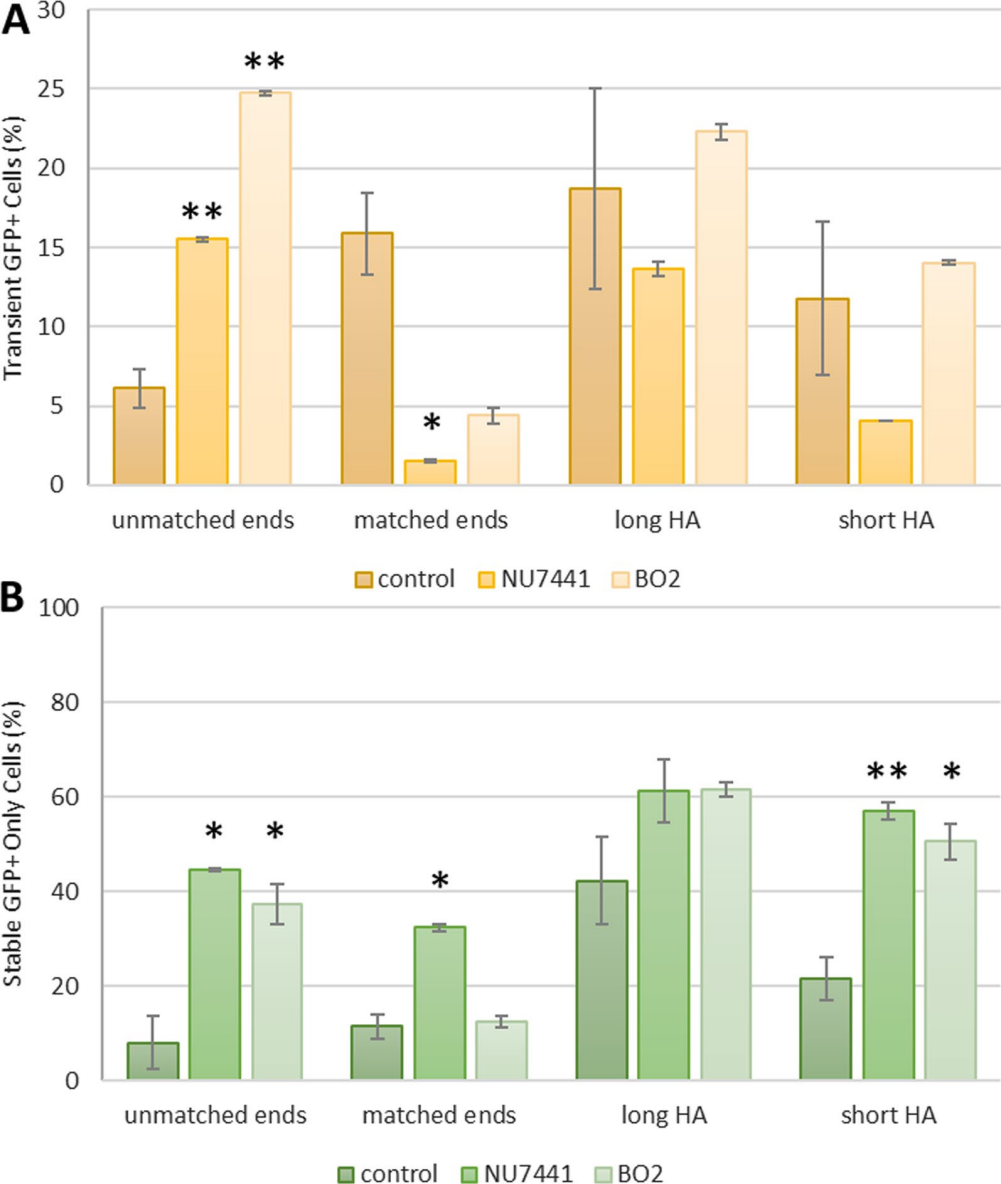
Inhibition of NHEJ or HR leads to small but significant increases in faithful integration of short HA donors. HEK293T cells were transfected with Cas9 and sgRNA plasmids along with the donor plasmid for either the unmatched ends, matched ends, long HA, or short HA strategy. **A** Cells were analysed for GFP+ only expression by flow cytometry 2 days after transfections. Error bars indicate standard error from three biological replicates. **B** Transfected cells were grown under puromycin selection for 3 weeks before final flow cytometry analysis. Error bars indicates standard error from ≥ two biological replicates. *p < 0.05; **p < 0.01 in post-hoc Tukey (Additional file 1: Tables S2 and S3)

When looking at the initial transgene integration efficiencies, both NU7441 and B02 led to a decrease in GFP+ cells with the matched ends strategy (Fig. 11). This indicates that NHEJ and HR can both participate in transgene integration when the transgene donor has free ends and homology to the integration site.

It was interesting that none of the inhibitors increased the fidelity of KI using the long homology arms strategy (Fig. 11). However, both NU7441 and B02 led to an increase in KI fidelity using the short HA strategy, whereas rucaparib completely abolished the survival of GFP+ cells in the short HA experiment. This indicates that MMEJ may be the predominant repair pathway for integration of the short HA donor.

### Cas9-mediated integration generates distinct KI outcomes in K562 compared to HEK293T

To evaluate whether integration outcomes are distinct in other cell types, we tested our assay in K562 cells. K562 cells carries the BCR-Abl fusion protein, which alters DNA damage response and repair process. Indeed, molecular studies on K562 cells demonstrated an upregulation of MMEJ activator proteins and downregulation of DNA-PK and DNA ligase IV suggesting the preferential of MMEJ against NHEJ in BCR-Abl cells [56–58]. We included matched end joining, long HA, short HA, short 5ʹ overhang and short 3ʹ overhang strategies with multiple Cas9 variants; Cas9, Cas9es, and Geminin-fused Cas9.

Interestingly, higher transgene integration efficiency was observed in K562 compared to HEK293 cells from all strategies using Cas9es (Table 3 and Additional file 1: Table S6). This suggested that a decrease in Cas9 off-target activity or re-cleavage activity, provided by the use of enhanced specificity Cas9 variants, results in improved transgene integration efficiency in BCR-Abl active cells.

**Table 3:** Strategies that produced a significant change in initial transgene integration efficiency in K562 compared to HEK293T

Strategy	Cas9 variant	Log2 fold differences
5′ overhang-pair 1	Cas9es	2.995068471
Long HA	Cas9es	2.434625651
Short HA	Cas9es	2.2101824
Matched ends	Cas9es	2.019033767
3′ overhang-pair 1	Cas9es	1.261492241
Short HA	Cas9-Gem	0.753938673
Long HA	Cas9-Gem	0.62552389
3′ overhang-pair 1	Cas9-Gem	− 0.889219569
3′ overhang-pair 1	Cas9es-Gem	− 1.073854847
5′ overhang-pair 1	Cas9	− 1.330448081
Matched ends	Cas9-Gem	− 1.469485283
Matched ends	Cas9	− 2.423440966
5′ overhang-pair 1	Cas9es	− 3.482663925
5′ overhang-pair 1	Cas9-Gem	− 4.743391863

Most strategies generated similar outcomes of faithful transgene integrations between cell lines. Surprisingly, 3ʹ overhang strategies generated higher fidelity in K562 cells compared to HEK293T cells (Table 4). This indicates that faithful repair processing of 3ʹ overhang intermediates will mostly rely on MMEJ repair pathways as opposed to other DNA repair pathways.

**Table 4:** Strategies that produced a significant change in faithful transgene integration in K562 compared to HEK293T cells

Strategy	Cas9 variant	Log2 fold differences
3′ overhang-pair 1	Cas9-Gem	1.496265155
3′ overhang-pair 1	Cas9es	0.901494278
5′ overhang-pair 1	Cas9es	− 1.431344377
Short HA	Cas9es-Gem	− 2.559634321

## Discussion

Reports on unwanted editing outcomes from CRISPR–Cas9 editing has risen in numbers recently [35–42]. This highlights the need for additional robust assays that could detect these unwanted events and to evaluate faithful editing outcomes. Here we have developed an assay that is able to assess CRISPR–Cas9 editing strategies in integrating transgene fragments while monitoring unwanted DNA integrations. We found that unwanted DNA integration, shown by DNA backbone integration, is prominent with transgene integration and tarnished the efficiency of integration (Figs. 1 and 2). With these findings, we proposed that fidelity of CRISPR–Cas9 editing should not only focus on the transgene and the target locus but also evaluate the absence of unwanted DNA integration in the genome.

This unwanted integration might be caused by different factors which involve the choice of CRISPR–Cas9 strategy. First, the choice of DNA repair pathways directly affects the editing outcomes. Cas9 strategies were designed by how DNA repair factors reacts and responds to DNA DSBs [8, 9, 12, 13]. End-joining strategies, which involve linearization of the donor plasmid, resulted in high unwanted backbone integration, despite homologous sequences on the transgene plasmid and the lack of homologous sequences on the backbone fragment (Fig. 2). This indicates that linearized DNA fragments are more likely to trigger the non-homologous repair pathway, and could not differentiate the transgene and the non-transgene DNA fragment as donor fragments upon integration.

Circulated donors will require DNA repair factors that invade the closed double strand template for DNA integrations. This could be achieved through HR and alt-EJ repair pathways, utilizing Rad51 and PARP1 proteins, respectively [54, 59]. Nevertheless, unwanted backbone fragment was also found in these strategies despite the presence of homologous sequences at both ends of the transgene (Fig. 3). This indicates that integration using HR-designed strategies might also be un-efficient possibly due to integration directed by one homologous arm where it integrates the whole plasmid on the target locus.

Furthermore, we tested end joining strategies with 5ʹ or 3ʹ overhang intermediate DNA DSBs using dual Cas9 nickase system (Fig. 4). A pair of sgRNA guides Cas9 nickases to target the AAVS1 locus as well as linearizing the donor plasmid and provide homologous sequence on the ssDNA overhangs. This will prime for direct end-to-end joining of the transgene to the cleaved locus. The different types and lengths of overhangs enable us to assess the impact of ssDNA homologous sequences on transgene integration.

Initial transgene integration efficiency utilizing the overhang DNA DSB strategies were lower compared to blunt end strategies (Additional file 1: Table S4). The lower integration efficiency might be due to the kinetics of paired Cas9 nickase in generating DNA DSBs in which it requires double targeting at both ends of the target [2, 11, 60, 61]. Furthermore, the ssDNA overhangs might recruit and initiate DNA end processing mechanism which prolong the time for repair and integration [62, 63].

The length of ssDNA overhangs influences the KI profile outcome (Fig. 6). The end formation of DNA DSBs could draw specific proteins and responses from the different DNA repair machineries [62, 63]. The long 5ʹ overhang strategy, generated by pair 5, and the short 3ʹ overhang strategy generated by pair 1, yield high GFP+BFP− cells in puromycin selected cells (Fig. 6). This implies that the long 5ʹ overhangs and the short 3ʹ overhangs could direct proper transgene recognition and integration whilst reducing unwanted backbone fragment integrations. Moreover, this suggested that the type and length of ssDNA overhangs may influence the DNA repair choices which in turn will impact the KI outcomes of CRISPR–Cas9 strategies.

Cas9 off-target activity generates DNA DSBs at off-target sites that could promote unwanted DNA integration. These events are challenging to evaluate as it would require thorough sequencing methods. With our assay, we found that enhanced specificity Cas9 variant alone did not increase integration efficiency nor fidelity (Figs. 3, 5, and 6). Kinetic studies have shown that Cas9es has similar on-target activity while reducing the off-target activity [47, 64]. This indicates that integration of transgene and non-transgene fragment is most likely on-target. Further genomic analysis is required to support this finding.

An increase in efficiency and fidelity was seen upon applying a cell-cycle regulated Cas9 expression system (Fig. 7). The addition of a minimal region of Geminin on the N-terminus of the Cas9 coding sequence was shown to limit Cas9 activity at the late S to M phase [32, 33]. This is desirable for HR editing as it is active during the S phase of the cell cycle. The increase in fidelity indicates that repair and integration processes in the S-M phase would be desirable for high fidelity integration outcomes. Restricting Cas9 activity to S phase when HR is active and limiting its off-target cleavage activity with the enhanced specificity mutations, results in more efficient on-target integration of the transgene and reduced integration of the plasmid backbone, which does not have homology arms.

Interestingly the use of Cas9es with geminin generated a significant difference in integration outcomes compared to conventional Cas9 (Fig. 7). This might indicate that the effect of off-target activity, while minimal in non-cell cycle regulated Cas9 activity, could be magnified during the S-M phase of the cell cycle.

Reducing Cas9 expression greatly improves the efficiency and fidelity of transgene integration (Fig. 10 and Additional file 1: Table S5). This could be indicative to reduce off-targeting activity or on-target transgene re-cleavage activity. Transgene fragment that was integrated on-target would reconstitute the target sequence for Cas9 targeting. As Cas9 is a multiple turnover enzyme, this would enable re-targeting of the transgene and reducing editing efficiency and fidelity. Limiting Cas9 by using mRNA and ribonucleoprotein (RNP) platforms was shown to increase faithful editing outcomes [25, 27, 29]. Our self-cleaving strategy with the DNA platform adds up to the choice of short-lived Cas9 platforms while maintaining the advantage of high-efficient DNA transfection.

By employing small molecules such as NU7441 and BO2 to inhibit DNA-PK and Rad51, respectively, we gain insights on how specific DNA repair pathways responds to a particular strategy and influence the editing outcomes. In our assay, DNA-PK inhibition and Rad51 inhibition led to a decrease in transgene integration efficiency and a slight increase in fidelity of matched-end joining strategy, respectively (Fig. 11). Interestingly, DNA repair pathway inhibition did not produce a significant effect on HR-based editing outcomes. This indicates that with circular donor plasmid integration could be integrated through multiple pathways and does not depend on HR machinery.

Upon comparing KI profiles in two distinct cell lines, HEK293T and K562, we observed variations in the outcomes of certain CRISPR–Cas9 strategies between them (Tables 3 and 4). The use of enhanced specificity Cas9 increases integration efficiency in BCR:Abl+ cells. Moreover, short 3ʹ overhang strategies produced higher transgene integration fidelity compared to HEK293T results (Table 4). This confirms that biological differences in DNA repair preferences between K562 and HEK293 cells would generate distinct outcomes.

Non-DSB mediated editing, namely base editing and prime editing, has been shown to generate efficient point editing with lower off-target activities (Reviewed by Anzalone et al. [65]). While promising for single base or short editing uses, these technologies are limited by the length of DNA sequences that could be integrated at each loci. Previously, it has been reported that a combination between prime editing and site-specific recombinase (SSR) could provide targeted integration of a transgene into a pre-installed SSR landing site [66]. However, this strategy would require multiple steps of editing compared to the CRISPR–Cas9 KI strategies. Particularly evident in our findings, improvements in large DNA integration fidelity were observed when employing various CRISPR–Cas9 editing strategies. Each variation in strategy may activate specific DNA repair pathway mechanisms, which must be considered when devising a CRISPR–Cas9-based gene editing approach.

## Conclusion

We reported the development of a novel gene integration assay that would enable robust and multiple evaluation of CRISPR–Cas9 KI strategies. With this assay we confirm that unwanted genetic integration of CRISPR–Cas9 editing happens prominently. Unsurprisingly, the fidelity of transgene integration is determined by multiple factors which is influenced by the choice of DNA repair pathway and Cas9 cleavage activity. Limiting Cas9 activity by reducing Cas9 expression or restricting activity to S-phase cell-cycle as well as utilizing a HR-based strategy was shown to generate editing outcomes with the highest efficiency and fidelity. Furthermore, our findings contribute to the current literature which indicates that CRISPR–Cas9 strategies trigger specific DNA repair mechanism, influencing integration efficiency and fidelity outcomes. This highlights the significance of understanding the cell’s preferred DNA repair mechanism to devise an optimal strategy for transgene integrations.

These results generated in HEK293T and K562 cell lines gave us a better understanding on how DNA integration outcomes are influenced by the CRISPR–Cas9 strategy. It would be vital to apply this assay in different cell lines and primary cells that have different DNA repair profiles to better understand the correlation between DNA repair choices to editing outcomes. Additionally, these findings could be better characterized in a genomic scale through thorough genomic sequencing on the target junctions as well as fluorescence hybridization to detect copy number and to locate any off-target integrations. Lastly, the use of Cas9 mRNA and RNP has been used as a gold standard for CRISPR–Cas9 delivery. It would be interesting to compare the results of using different platforms of CRISPR–Cas9 delivery vectors to integration outcomes in in vitro as well as in vivo settings.

### Supplementary Information

**Additional file 1: Figure S1.** Density plot of puromycin-resistant cells gated depending on GFP and BFP fluorescence. B−/G− indicates cell population that does not have blue nor green fluorescence; B+/G+ indicates cell population that has both blue and green fluorescence; Blue+ indicates cell population that expresses blue fluorescence only; and Green+ indicates cell population that expresses green fluorescence only. **Table S1.** AAVS1 pair guides and overhang lengths. **Table S2.** p-values of unselected GFP+ only cells in Fig. 11A Inhibition of NHEJ or HR leads to small but significant increases in faithful integration of short HA donors. n.s.: statistically not significant. **Table S3.** p-values of stable GFP+ only cells Fig. 11B Inhibition of NHEJ or HR leads to small but significant increases in faithful integration of short HA donors**.** n.s.: statistically not significant. **Table S4.** HEK 293T KI outcomes. **Table S5.** HEK 293T KI outcomes of self-cleaving results. **Table S6.** K562 KI outcomes.

## Data Availability

Data and materials used in this study are available from the corresponding author on request.

## Abbreviations

AAVS1: Adeno-associated virus locus 1
BFP: Blue fluorescent protein
CRISPR: Clustered regularly interspaced palindromic repeat
Cas9: CRISPR-associated protein 9
FACS: Fluorescent-activating cell sorting
GFP: Green fluorescent protein
HR: Homologous recombination
KI: Knock-in
NHEJ: Non-homologous end joining
MMEJ: Microhomology-mediated end joining
sgRNA: Single guide RNA
