# Supplementary material for: Improving CRISPR–Cas9 directed faithful transgene integration outcomes by reducing unwanted random DNA integration
Source: J Biomed Sci. 2024 Mar 26;31:32. doi: 10.1186/s12929-024-01020-x (PMC10964699; doi:10.1186/s12929-024-01020-x)
Supplement: Supplementary file 1 — Additional file 1: Figure S1. Density plot of puromycin-resistant cells gated depending on GFP and BFP fluorescence. B−/G− indicates cell population that does not have blue nor green fluorescence; B+/G+ indicates cell population that has both blue and green fluorescence; Blue+ indicates cell population that expresses blue fluorescence only; and Green+ indicates cell population that expresses green fluorescence only. Table S1. AAVS1 pair guides and overhang lengths. Table S2. p-values of unselected GFP+ only cells in Fig. 11A Inhibition of NHEJ or HR leads to small but significant increases in faithful integration of short HA donors. n.s.: statistically not significant. Table S3. p-values of stable GFP+ only cells Fig. 11B Inhibition of NHEJ or HR leads to small but significant increases in faithful integration of short HA donors. n.s.: statistically not significant. Table S4. HEK 293T KI outcomes. Table S5. HEK 293T KI outcomes of self-cleaving results. Table S6. K562 KI outcomes. [file 12929_2024_1020_MOESM1_ESM.docx]

# Supplementary Information

**Improving CRISPR-Cas9 directed faithful transgene integration outcomes by reducing unwanted random DNA integration**

Plasmid maps could be accessed through this link: <https://drive.google.com/drive/folders/1oudE9OwLvMfrxisGmUWtXTGNGFnEgme8?usp=drive_link>

**Figure S1.** Density plot of puromycin-resistant cells gated depending on GFP and BFP fluorescence. B-/G- indicates cell population that does not have blue nor green fluorescence; B+/G+ indicates cell population that has both blue and green fluorescence; Blue+ indicates cell population that expresses blue fluorescence only; and Green+ indicates cell population that expresses green fluorescence only.

**Table S1** AAVS1 pair guides and overhang lengths

| AAVS pair guides | | | |
| --- | --- | --- | --- |
| Pairs | Guide 1 | Guide 2 | Overhang length |
| Pair 1 | A1 | B2 | 31 bp |
| Pair 2 | A1 | B3 | 61 bp |
| Pair 3 | A2 | B1 | 67 bp |
| Pair 4 | A3 | B1 | 106 bp |
| Pair 5 | A2 | B3 | 121 bp |

**Table S2.** p-values of unselected GFP+ only cells in **Figure 11A** Inhibition of NHEJ or HR leads to small but significant increases in faithful integration of short HA donors**.** n.s.: statistically not significant.

| **Unmatched ends** | | | |
| --- | --- | --- | --- |
|  | Control | NU7441 | BO2 |
| Control |  | 0.005399 | 0.001005 |
| Nu7441 | 0.005399 |  | 0.008007 |
| BO2 | 0.001005 | 0.008007 |  |
| **Matched ends** | | | |
|  | Control | NU7441 | BO2 |
| Control |  | 0.037946 | n.s. |
| Nu7441 | 0.037946 |  | n.s. |
| BO2 | n.s. | n.s. |  |

**Table S3.** p-values of stable GFP+ only cells **Figure 11B** Inhibition of NHEJ or HR leads to small but significant increases in faithful integration of short HA donors**.** n.s.: statistically not significant.

| **Unmatched ends** | | | |
| --- | --- | --- | --- |
|  | Control | NU7441 | BO2 |
| Control |  | 0.0127574 | 0.027746 |
| Nu7441 | 0.0127574 |  | n.s. |
| BO2 | 0.0277461 | n.s. |  |
| **Matched ends** | | | |
|  | Control | NU7441 | BO2 |
| Control |  | 0.0175824 | n.s. |
| Nu7441 | 0.0175824 |  | n.s. |
| BO2 | n.s. | n.s. |  |
| **Short HA** | | | |
|  | Control | NU7441 | BO2 |
| Control |  | 0.0078065 | 0.016174 |
| Nu7441 | 0.0078065 |  | n.s. |
| BO2 | 0.0161738 | n.s. |  |

**Table S4**. HEK 293T KI outcomes

| Strategy | Cas9 variants | Unselected cells | | | Puromycin stable cell | | | | |
| --- | --- | --- | --- | --- | --- | --- | --- | --- | --- |
|  |  | Ame | GFP+ | sem GFP+ | GFP+ | GFP+/BFP+ | BFP+ | unselected | sem GFP+BFP- |
| random end joining | Cas9 | 53.80 | 10.05 | 3.68 | 9.00 | 82.46 | 3.22 | 5.33 | 4.09 |
|  | Cas9es | 53.20 | 6.28 | 1.43 | 18.90 | 72.55 | 4.03 | 4.52 | 6.43 |
|  | Cas9-Gem | 44.30 | 36.67 | 0.87 | 31.30 | 61.53 | 5.87 | 1.30 | 9.31 |
|  | Cas9es-Gem | 67.87 | 18.77 | 0.64 | 36.40 | 4.07 | 59.07 | 0.47 | 4.65 |
| matched ends | Cas9 | 61.06 | 15.87 | 2.57 | 9.60 | 76.09 | 12.79 | 1.52 | 2.27 |
|  | Cas9es | 54.72 | 16.64 | 2.13 | 12.88 | 71.27 | 12.98 | 2.88 | 3.23 |
|  | Cas9-Gem | 45.73 | 6.00 | 0.54 | 6.00 | 72.33 | 19.83 | 1.83 | 0.25 |
|  | Cas9es-Gem | 50.17 | 15.37 | 2.19 | 8.00 | 79.53 | 10.90 | 1.57 | 0.74 |
| Long HA | Cas9 | 62.10 | 18.70 | 6.30 | 24.21 | 56.20 | 15.85 | 3.75 | 0.91 |
|  | Cas9es | 43.70 | 5.40 | 1.34 | 32.67 | 57.74 | 6.81 | 2.79 | 7.45 |
|  | Cas9-Gem | 49.67 | 15.47 | 0.44 | 58.67 | 14.33 | 1.07 | 25.93 | 0.24 |
|  | Cas9es-Gem | 55.13 | 27.73 | 1.77 | 77.03 | 8.30 | 0.03 | 14.63 | 0.09 |
| Short HA | Cas9 | 63.14 | 11.79 | 4.86 | 21.82 | 64.03 | 8.75 | 5.40 | 4.78 |
|  | Cas9es | 40.55 | 4.85 | 0.96 | 28.49 | 56.90 | 8.31 | 6.31 | 6.95 |
|  | Cas9-Gem | 41.27 | 3.38 | 0.21 | 17.03 | 71.20 | 9.00 | 2.77 | 1.15 |
|  | Cas9es-Gem | 52.33 | 24.03 | 1.38 | 48.93 | 30.67 | 5.67 | 14.73 | 2.50 |
| D10A P1 | Cas9 | 53.75 | 5.40 | 0.89 | 37.45 | 59.13 | 2.53 | 0.89 | 10.13 |
|  | Cas9es | 47.37 | 4.52 | 0.61 | 26.33 | 67.96 | 2.46 | 3.25 | 3.43 |
|  | Cas9-Gem | 62.66 | 5.95 | 0.41 | 14.27 | 70.23 | 13.70 | 1.80 | 3.35 |
|  | Cas9es-GEM | 52.07 | 19.23 | 0.64 | 15.57 | 71.73 | 9.83 | 2.87 | 3.23 |
| D10A P2 | Cas9 | 58.07 | 5.36 | 1.36 | 19.26 | 62.73 | 10.90 | 7.11 | 11.91 |
|  | Cas9es | 44.03 | 3.21 | 0.79 | 36.23 | 54.10 | 6.25 | 3.42 | 14.92 |
| D10A P3 | Cas9 | 66.02 | 6.89 | 1.33 | 19.71 | 70.01 | 3.02 | 7.26 | 10.23 |
|  | Cas9es | 47.25 | 4.40 | 1.35 | 34.67 | 59.97 | 2.71 | 2.65 | 5.16 |
| D10A P4 | Cas9 | 68.56 | 5.01 | 1.65 | 29.56 | 64.58 | 4.03 | 1.82 | 6.17 |
|  | Cas9es | 40.70 | 4.21 | 1.35 | 31.88 | 60.64 | 2.13 | 5.36 | 11.41 |
| D10A P5 | Cas9 | 66.32 | 4.31 | 1.59 | 61.13 | 35.48 | 1.63 | 1.76 | 2.72 |
|  | Cas9es | 43.50 | 4.63 | 0.71 | 32.70 | 60.65 | 3.40 | 3.25 | 6.61 |
|  | Cas9-Gem | 62.35 | 6.25 | 0.32 | 36.47 | 51.50 | 6.47 | 5.57 | 5.81 |
|  | Cas9es-Gem | 36.20 | 22.10 | 0.44 | 37.83 | 49.60 | 7.60 | 4.97 | 3.40 |
| H840A P1 | Cas9 | 54.81 | 5.02 | 0.70 | 44.64 | 50.56 | 1.85 | 2.95 | 7.72 |
|  | Cas9es | 48.99 | 5.72 | 0.69 | 47.98 | 41.19 | 2.66 | 8.17 | 8.65 |
|  | Cas9-Gem | 61.15 | 6.39 | 0.32 | 34.10 | 57.30 | 6.77 | 1.83 | 3.09 |
|  | Cas9es-Gem | 52.07 | 17.37 | 0.37 | 37.43 | 54.23 | 4.40 | 3.93 | 3.32 |
| H840A P2 | Cas9 | 59.76 | 5.72 | 1.17 | 24.71 | 66.77 | 4.84 | 3.69 | 11.23 |
|  | Cas9es | 43.28 | 5.86 | 1.90 | 33.86 | 58.02 | 3.64 | 4.48 | 4.63 |
| H840A P3 | Cas9 | 70.00 | 7.75 | 2.34 | 23.81 | 68.90 | 5.04 | 2.25 | 9.53 |
|  | Cas9es | 56.50 | 4.39 | 1.68 | 32.74 | 51.74 | 3.66 | 11.86 | 12.36 |
| H840A P4 | Cas9 | 52.03 | 5.26 | 2.87 | 37.40 | 58.10 | 2.30 | 2.20 | 0.10 |
|  | Cas9es | 51.18 | 3.74 | 1.00 | 41.85 | 52.90 | 1.82 | 3.44 | 16.24 |
| H840A P5 | Cas9 | 69.78 | 8.54 | 2.80 | 32.50 | 61.35 | 2.60 | 3.55 | 6.28 |
|  | Cas9es | 60.03 | 4.94 | 1.74 | 35.66 | 57.95 | 3.37 | 3.03 | 9.74 |
|  | Cas9-Gem | 60.27 | 5.91 | 0.12 | 53.53 | 38.17 | 4.70 | 3.60 | 1.33 |
|  | Cas9es-Gem | 31.87 | 19.90 | 1.39 | 59.37 | 31.73 | 1.73 | 7.17 | 6.36 |

**Table S5**. HEK 293T KI outcomes of self-cleaving results

| Strategy | Cas9 variants | Unselected cells | | | Puromycin stable cell | | | | |
| --- | --- | --- | --- | --- | --- | --- | --- | --- | --- |
|  |  | Ame | GFP+ | sem GFP+ | GFP+ | GFP+/BFP+ | BFP+ | unselected | sem GFP+BFP- |
| matched ends | Cas9 | 48.03 | 54.47 | 0.57 | 14.43 | 83.80 | 1.23 | 0.53 | 0.84 |
|  | Cas9es | 50.67 | 57.33 | 1.49 | 18.67 | 78.30 | 1.50 | 1.53 | 0.64 |
|  | Cas9-Gem | 56.10 | 59.97 | 0.38 | 27.60 | 70.47 | 0.83 | 1.10 | 1.51 |
|  | Cas9es-Gem | 51.67 | 55.30 | 2.63 | 33.90 | 63.73 | 0.77 | 1.60 | 1.56 |
| Long HA | Cas9 | 41.90 | 65.37 | 0.55 | 81.10 | 13.87 | 0.00 | 5.03 | 0.50 |
|  | Cas9es | 52.63 | 68.80 | 1.19 | 78.27 | 14.30 | 0.00 | 7.43 | 0.88 |
|  | Cas9-Gem | 54.37 | 66.50 | 0.29 | 80.70 | 14.13 | 0.00 | 5.17 | 0.30 |
|  | Cas9es-Gem | 53.63 | 64.37 | 1.26 | 78.93 | 15.47 | 0.00 | 5.60 | 0.48 |
| Short HA | Cas9 | 52.13 | 64.93 | 0.44 | 29.77 | 63.00 | 1.93 | 5.30 | 0.32 |
|  | Cas9es | 66.23 | 57.03 | 1.76 | 39.50 | 55.47 | 1.60 | 3.43 | 1.15 |
|  | Cas9-Gem | 63.50 | 60.83 | 0.57 | 38.43 | 56.97 | 1.60 | 3.00 | 1.58 |
|  | Cas9es-Gem | 45.63 | 61.47 | 0.22 | 32.77 | 57.87 | 1.80 | 7.57 | 1.28 |
| Short 5’ overhang end joining | Cas9 | 16.97 | 18.37 | 0.38 | 30.67 | 56.03 | 8.83 | 4.47 | 2.78 |
|  | Cas9es | 31.63 | 37.23 | 0.79 | 56.50 | 33.63 | 4.07 | 5.80 | 5.55 |
|  | Cas9-Gem | 39.17 | 28.83 | 0.84 | 33.33 | 53.07 | 6.93 | 6.67 | 4.83 |
|  | Cas9es-Gem | 33.67 | 41.03 | 1.82 | 41.27 | 46.17 | 5.70 | 6.87 | 4.32 |
| Long 5’ overhang end joining | Cas9 | 32.50 | 26.17 | 0.64 | 42.10 | 46.17 | 7.37 | 4.37 | 2.29 |
|  | Cas9es | 34.17 | 25.67 | 0.18 | 34.57 | 52.73 | 7.27 | 5.43 | 1.73 |
|  | Cas9-Gem | 35.57 | 21.03 | 1.11 | 47.67 | 39.90 | 5.60 | 6.83 | 2.29 |
|  | Cas9es-Gem | 37.93 | 21.80 | 0.80 | 39.77 | 42.57 | 6.60 | 11.07 | 4.69 |
| Short 3’ overhang end joining | Cas9 | 51.10 | 3.23 | 0.09 | 24.23 | 73.47 | 1.30 | 1.00 | 4.79 |
|  | Cas9es | 58.63 | 0.80 | 0.00 | 28.00 | 68.10 | 3.57 | 0.33 | 6.15 |
|  | Cas9-Gem | 57.73 | 5.03 | 0.19 | 34.57 | 63.67 | 0.50 | 1.27 | 6.41 |
|  | Cas9es-Gem | 60.33 | 1.10 | 0.06 | 33.70 | 62.83 | 1.90 | 1.57 | 5.51 |
| Long 3’ overhang end joining | Cas9 | 61.50 | 3.10 | 0.06 | 43.60 | 52.67 | 3.40 | 0.33 | 15.21 |
|  | Cas9es | 61.57 | 1.10 | 0.12 | 12.70 | 77.70 | 9.00 | 0.60 | 1.70 |
|  | Cas9-Gem | 62.67 | 2.33 | 0.03 | 62.43 | 26.30 | 4.33 | 6.93 | 11.99 |
|  | Cas9es-Gem | 66.47 | 1.30 | 0.06 | 53.63 | 45.83 | 0.23 | 0.30 | 17.75 |

**Table S6**. K562 KI outcomes

| Strategy | Cas9 variants | Unselected cells | | | Puromycin stable selected cells | | | | |
| --- | --- | --- | --- | --- | --- | --- | --- | --- | --- |
|  |  | Ame | GFP+ | sem GFP | GFP+ | BFP+ | GFP+/BFP+ | unselected | sem GFP+ |
| matched end joining | Cas9 | 54.05 | 3.27 | 1.47 | 28.43 | 1.53 | 69.87 | 0.17 | 11.09 |
|  | Cas9es | 87.90 | 66.63 | 2.78 | 19.63 | 0.00 | 80.27 | 0.10 | 10.13 |
|  | Cas9-gem | 52.33 | 2.17 | 0.07 | 33.13 | 16.93 | 47.13 | 2.80 | 13.59 |
|  | Cas9es-gem | 61.03 | 7.80 | 0.45 | 16.93 | 0.10 | 82.80 | 0.17 | 6.57 |
| long HA | wt | 67.07 | 33.23 | 2.54 | 30.93 | 0.00 | 51.93 | 17.13 | 3.74 |
|  | es | 65.27 | 29.17 | 0.88 | 34.97 | 1.33 | 45.77 | 17.93 | 14.91 |
|  | wt-gem | 33.37 | 23.87 | 1.36 | 43.73 | 3.50 | 51.87 | 0.90 | 5.42 |
|  | es-gem | 70.20 | 31.27 | 2.27 | 64.30 | 0.00 | 19.00 | 16.70 | 16.73 |
| short HA | wt | 46.60 | 20.95 | 4.65 | 40.10 | 0.80 | 31.40 | 27.70 | 30.50 |
|  | es | 51.30 | 22.43 | 0.61 | 46.17 | 0.40 | 51.03 | 2.40 | 22.48 |
|  | wt-gem | 54.37 | 5.70 | 0.55 | 14.90 | 71.07 | 13.67 | 0.37 | 6.77 |
|  | es-gem | 49.30 | 15.93 | 2.79 | 8.30 | 35.83 | 53.50 | 2.37 | 2.94 |
| Short 5 overhang end joining | wt | 42.77 | 2.17 | 0.43 | 17.37 | 6.83 | 73.80 | 2.00 | 2.85 |
|  | es | 44.43 | 34.27 | 1.29 | 10.67 | 0.63 | 88.23 | 0.47 | 4.96 |
|  | wt-gem | 60.65 | 1.25 | 0.15 | 19.40 | 1.65 | 77.35 | 1.60 | 2.80 |
| Long 5 overhang end joining | wt | 76.57 | 0.90 | 0.17 | 61.37 | 0.07 | 37.80 | 0.77 | 2.74 |
|  | es | 26.45 | 0.35 | 0.05 | 14.40 | 0.00 | 85.20 | 0.40 | 1.70 |
|  | wt-gem | 59.17 | 0.23 | 0.12 | 26.27 | 0.07 | 73.30 | 0.37 | 1.71 |
|  | es-gem | 47.80 | 10.85 | 1.65 | 38.10 | 0.00 | 61.80 | 0.10 | 11.00 |
| Short 3 overhang end joining | wt | 55.60 | 8.60 | 3.00 | 59.75 | 0.65 | 39.20 | 0.40 | 16.65 |
|  | es | 84.95 | 12.75 | 1.35 | 97.90 | 0.00 | 1.75 | 0.35 | 1.00 |
|  | wt-gem | 77.60 | 3.45 | 0.35 | 96.20 | 0.20 | 3.30 | 0.30 | 0.40 |
|  | es-gem | 83.75 | 8.25 | 0.15 | 69.75 | 0.00 | 30.15 | 0.10 | 24.35 |
